# Implementation of universal newborn bloodspot screening for sickle cell disease and other clinically significant haemoglobinopathies in England: screening results for 2005–7

**DOI:** 10.1136/jcp.2008.058859

**Published:** 2008-12-15

**Authors:** A Streetly, R Latinovic, K Hall, J Henthorn

**Affiliations:** 1 NHS Sickle Cell and Thalassaemia Screening Programme, King’s College London School of Medicine, Division of Health and Social Care Research, London, UK; 2 Clinical Chemistry Department, Birmingham Children’s Hospital NHS Trust, Birmingham, UK; 3 Central Middlesex Hospital, North West London Hospitals NHS Trust, London, UK

## Abstract

Early results from the National Health Service Sickle Cell and Thalassaemia Screening programme covering the whole of England are reported following the implementation of the national newborn blood-spot screening programme. Of the 13 laboratories performing screening, 10 chose high-performance liquid chromatography as the first screen, with isoelectric focusing as the second confirmatory test. Screening results for April 2005 to March 2007 are presented and include data from all the laboratories screening newborns in England, and almost 1.2 million infants. The screen-positive results show a national birth prevalence of almost 1 in 2000. The birth prevalence in London is five times that of most of the rest of the country. Over 17 000 carriers have been identified. Approximately seven per 1000 samples are reported as post-transfusion with wide ethnic category variation. Given the prevalence of the conditions, and coverage by ethnicity, 3–4 screen-positive cases could be missed each year. National implementation of newborn screening in England has increased the number of children identified with sickle cell disease, in many areas almost doubling the workload. Underascertainment of the condition has allowed a downplaying of the scale of need. It may also have contributed to infant mortality rates in urban areas as babies died without a diagnosis or treatment. The value of a co-ordinated national approach to policy development and implementation is emphasised by the English experience. The programme provides a model for Europe as well as other countries with significant minority populations, such as Canada. Potentially it also offers important lessons for Africa where the World Health Organization is supporting the introduction of newborn screening.

In England the National Health Service (NHS) Plan published in 2000 gave a commitment to implement a linked antenatal and newborn haemoglobinopathy and sickle cell disease (SCD) screening programme by 2004.^1^ This was the culmination of many years of lobbying and reports making the case for screening of newborns.^2^^–^4 It followed consideration by, and support of, the Children’s Sub-group of the UK National Screening Committee (NSC) from the evidence of two Health Technology Assessments on the subject of newborn and antenatal screening for haemoglobinopathies.^5^ ^6^ These Health Technology Assessments supported the introduction of newborn screening for SCD using a mixture of universal and targeted approaches according to the prevalence of the condition.^5^ ^6^

Working within this policy framework, and incorporating advice from an international workshop held in 2001, the NHS Sickle Cell and Thalassaemia Screening Programme recommended to the Children’s Sub-group of the NSC that newborn screening offered to all babies, be recommended as the only practical and equitable solution to adopt.^7^ This recommendation was accepted in February 2002.

Only those haemoglobin (Hb) gene combinations that are clinically significant were specified for inclusion in the national screening programme. They are: HbSS, HbSC, HbSD-Punjab, HbS β thalassaemia, HbSO-Arab and HbSHPFH (hereditary persistence of fetal Hb).

The programme also recommended that other clinically significant conditions should be referred for clinical follow-up. These were conditions that did not meet the criteria for screening but were clinically significant and were likely to be detected by the methods used.^8^ ^9^ These are: β thalassaemia major, β thalassaemia intermedia, HbH disease, HbE/β thalassaemia and HbSE.^10^ The UK NSC recommended that the programme should not aim to identify combinations that are not clinically significant since there is no evidence that their detection leads to any benefit, and detection may, in fact, lead to harm through unnecessary medicalisation.^9^ The NSC supported the recommendation by the programme that, in line with pre-existing practice in areas already undertaking newborn screening, carriers of the main haemoglobins including S, C, D and E should be reported to parents. This was a controversial area, with many clinical geneticists considering that this information should be withheld.^11^ The UK decision is in line with the recent review undertaken in the USA.^12^

## METHODS

To prepare for national roll-out it was necessary to assess the readiness of the health service for implementation. An initial survey of health authorities and trusts undertaken in 2001 to assess readiness for implementation and support for implementation showed that there were significant challenges in terms of attitudes and lack of support from policy makers, health authorities and service providers for the programme.^13^ There was stronger support for the programme in higher prevalence areas although this was not completely consistent. The survey also suggested that laboratory policies (existing guidance) and services did provide a basis on which to build.^13^

To support the implementation of the programme, regional mapping exercises were undertaken for all regions of the country to attempt to identify care pathways for the care of affected children where these existed, or to highlight the need for these to be developed where they did not exist. These reports are all available on the programme website including flow-charts showing the care pathways that existed at the start of the programme.^14^ In some areas there was no such care pathway and no arrangements for communication of carrier results.

In addition, a survey of existing practice among the four laboratories already screening using dried blood spots showed that the majority employed high-performance liquid chromatography (HPLC) followed by isoelectric focusing (IEF) for confirmation, with one laboratory using IEF with no immediate confirmatory test. There was wide variation in the cost per test charged by commercial companies to these laboratories and to standardise quality and prices, national procurement was undertaken. This obviated the necessity for individual laboratories to go out to tender for equipment and ensured that equipment selected was the most suitable for the screening protocol chosen. In particular, automated HPLC equipment from the USA needed to be adapted for the UK market to take account of the different age at which testing is currently performed in the UK (5–8 days compared with earlier in the USA and elsewhere). The equipment had previously not been available in Europe.

To support the development of policy, various subgroups were established, including a laboratory subgroup and a training and education subgroup. The laboratory subgroup drew representation from the various relevant professionally led organisations and societies, as well as the recognised experts in the field, both clinicians and scientists. From this a plan for implementation was developed for each region or catchment area covered by a laboratory. The programme required the development and implementation of training for front-line staff involved with the programme, particularly midwives, and for this training to have been undertaken before implementation could proceed. The programme also liaised with a major quality assurance (QA) scheme and worked closely to support the development of a bespoke quality assurance for newborn screening based on dried blood spots to run alongside the existing liquid sample scheme.^15^ Most areas of policy are now clarified but work is ongoing to develop a consensus regarding unidentified variants and Hb Bart’s reporting, emphasising the need for better data on costs and benefits and further research.

The methods recommended by the programme were for an initial screening test using either HPLC or IEF to be performed with the biochemical screening already taking place for phenylketonuria and hypothyroidism. For initial screen-positive tests, a confirmatory test using a different technique was recommended. The decision to use two-stage testing to minimise incorrect identification of abnormal haemoglobins and ensure a high level of quality was based on the experience of the UK laboratories already using this approach and the USA, which had not routinely used a second test.^7^ The increase in workload implied by the roll-out for the West Midlands laboratory who were screening 15 000, and would increase to approximately 70 000 per annum, prompted an evaluation of available methodologies and the potential of each for automation. A primary focus was the potential of the technology to permit positive specimen identification (blood-spot card accession number) to follow a baby’s blood spot electronically from the punched disk to the analytical result.

In addition to providing start-up funding for laboratory development, funding for the development of adequate counselling services for affected infants and carriers identified by the programme was provided. All trusts were required to nominate a paediatrician to be the named local contact; this was especially important and difficult in lower prevalence areas where most care would be undertaken by a specialist unit distant from the local area. Materials to support communication with parents included a general “antenatal and newborn leaflet covering all screening including bloodspot screening” giving information on sickle cell disease^16^ and two carrier leaflets – one for HbS carriers^17^ and one for carriers of other haemoglobins.^18^

Implementation in the laboratories was supported by the production of a handbook^10^ detailing reporting formats and referral pathways. Ten standards of laboratory practice were produced covering accreditation, turnaround times and QA performance, and annual data returns were required to help monitor coverage and pick-up rates. We are currently working on status codes to relay information to the child health network and assist linkage with the antenatal programme. The programme has set national standards and is developing a QA programme covering all aspects of the screening and care pathway.^19^ ^20^

Planning of service capacity was based on epidemiological estimates provided by Professor Bernadette Modell.^21^ This gave estimates by area of the number of affected infants and carriers likely to be identified to ensure that resources and staffing were appropriate for the numbers expected.

These initial estimates were lower than the numbers from the early stages of implementation of screening. Subsequent estimates have been incorporated into the materials for the programme-funded national training programme called the “PEGASUS programme” for which Modell provided the public health module that incorporates estimates of need for newborn and antenatal screening. The other two modules of this programme were cascade training of trainers and materials to support “front line professionals” and specialist training for counsellors.^22^ Implementation was planned in four phases. A phased approach was adopted for several reasons. First, some high-prevalence areas, mainly in London, were already offering screening, and the programme wanted to build on the experience that already existed. Second, the policy was to implement screening universally based on the blood-spot programme already in place for newborn phenylketonuria and hypothyroidism screening. Prior to the implementation of sickle screening there were 20 biochemical laboratories with workloads ranging from 6000 to 100 000 specimens per annum. Information from a Health Technology Assessment report suggested that laboratories should cover at least 25 000 samples a year to be cost effective.^5^ This meant a reduction in the number of laboratories to 13, and this required commissioning engagement and reorganisation of services, and inevitably delayed implementation in some areas. In some areas “targeted screening” using cord blood methods needed to be discontinued to avoid duplication of effort and this was not always readily accepted.

The extent to which the managers in the NHS engaged with this process was significantly affected by a lack of coherence of government policy partly due to a major reorganisation of the NHS at this time and a lack of clarity as to where the responsibility for commissioning screening services lay—with arguments in many areas between local and regional commissioners and lack of ownership making securing the pick-up of funding following the start of the programme a significant challenge. These issues are now resolved but did delay implementation to some degree. Implementation in England started in September 2003 and was completed in July 2006. Implementation is planned for Scotland by 2010 but no date has yet been set for implementation in Wales and Northern Ireland.

## RESULTS

Of the 13 laboratories now undertaking newborn sickle cell screening 10 chose HPLC as the first screen, with IEF as the second confirmatory test. This second test is generally referred to laboratories with haematological expertise—either on-site or elsewhere.

Findings of the early stages of implementation prior to the complete introduction of the programme have previously been published.^23^ Screening results for the 2 years from April 2005 to March 2007 are shown in table 1. This provides almost complete information for the whole country (with the exception that Portsmouth provided data from April 2006, and Oxford from July 2006.). It includes reports from all the laboratories screening newborns in England, covering almost 1.2 million infants. Significant conditions comprise the following results: FS, FSC, FS other and FE (see supplementary table for detailed information on screening results and diagnostic possibilities). The supplementary table shows that the birth prevalence of screen-positive results—as listed in the boxes for the major conditions—shows a national birth prevalence of just above one in 2000. More than 300 screen-positive results are detected by newborn screening each year. The table also shows that birth prevalence in London is five times that of most of the rest of the country.

**Table 1:** Rates of significant conditions* per 1000 screened babies: April 2005 to March 2007

Results for newborns: April 2005 to March 2007	Significant conditions*		No. of babies screened
	Rate per 1000 babies screened	No.	
North East	0.12	6	49 321
North West	0.24	40	169 498
Yorkshire and The Humber	0.24	29	119 616
East Midlands	0.25	24	96 876
West Midlands	0.28	38	133 905
East of England	0.32	39	123 794
London	1.82	436	239 654
South East coast	0.14	14	97 954
South central	0.21	10	47 835
South West	0.12	11	90 520
Unknown		<5	29 641
England	0.54	651	1 198 614

*Significant conditions comprise the following results: FS, FSC, FS other conditions, and FE.

Over 17 000 carriers have been identified in the 2 years. Table 2 shows that over half of all carriers are identified in London but significant numbers of carriers are also being reported from the North West (includes Manchester), West Midlands (includes Birmingham), East of England Region (includes Essex) and East Midlands (includes Leicester and Nottingham). Table 3 shows that approximately seven per 1000 samples are reported as post-transfusion with wide ethnic category variation. Given the prevalence of the conditions, and coverage by ethnicity this is likely to represent 3–4 screen-positive cases missed each year.^24^ As a result of implementing haemoglobinopathy screening in the newborn period two cases where adult not newborn blood was on the card have also been identified; this is relevant for all the conditions screened for. An associated difficulty for the programme is the follow-up of premature infants.

**Table 2:** Carrier* rates per 1000 babies screened: April 2005 to March 2007

Results for newborns: April 2005 to March 2007	Carrier		No. of babies screened
	Rate per 1000 babies screened	No.	
North East	4.26	210	49 321
North West	7.89	1338	169 498
Yorkshire and The Humber	8.27	989	119 616
East Midlands	8.37	811	96 876
West Midlands	12.63	1691	133 905
East of England	10.18	1260	123 794
London	39.92	9566	239 654
South East coast	6.01	589	97 954
South central	8.30	397	47 835
South West	4.49	406	90 520
Unknown	3.91	116	29 641
England	14.49	17373	1 198 614

*Carrier status comprise the following results: FAS, FAC, FAD, FAE and other carrier.

**Table 3:** Transfused rates per 1000 babies screened: April 2005 to March 2007

Results for newborns: April 2005 to March 2007	Transfusions		No. of babies screened
	Rate per 1000 screened babies	No.	
White British	5.35	3554	664 710
White Irish	10.24	20	1953
Any other white background	5.33	214	40 126
White and black Caribbean	8.88	91	10 246
White and black African	5.83	32	5493
White and Asian	5.89	54	9164
Any other mixed background	7.23	115	15 912
Indian	6.67	165	24 744
Pakistani	6.55	211	32 214
Bangladeshi	6.83	100	14 649
Any other Asian background	7.24	85	11 738
Black Caribbean	16.62	208	12 512
Black African	12.87	524	40 728
Any other black background	16.56	55	3322
Chinese	11.20	54	4821
Any other ethnic category	8.58	215	25 051
Not stated	12.74	1679	131 828
England	7.03	7376	1 049 211

Birmingham was unable to provide transfused data due to variations in coding of ethnic category and laboratory software constraints and Bristol was unable to provide transfused data for the first half of 2005. Total numbers were reduced accordingly.

The West Midlands evaluation reviewed four systems: Akron IEF from Perkin Elmer, Primus Corporation HPLC, Bio-Rad Variant and Bio-Rad NHS (later known as NBS) HPLC systems. The main findings was that the Bio-Rad NBS HPLC system, which had been developed for the California State Newborn Screening Service, permitted full positive specimen identification, the crispest chromatograms and large workloads using a multiple microtitre plate format. Akron IEF, with excellent haemoglobin discrimination, was a close second in respect of automation. The algorithms in the HPLC software also needed amending to fit England’s later age of screening specimen; specifically around the amount of HbA present. This work informed the implementation.

More detailed information by local area, type of condition and carrier status will be available in due course. This will allow an update of the modelling data used to estimate expected numbers of carriers and disease states.

## DISCUSSION

National implementation of newborn screening in England has increased the number of children identified with SCD, in many areas almost doubling the workload over a few years. This shows how underascertainment of the condition has in the past allowed a downplaying of the needs of people with these conditions. It may also have contributed to infant mortality rates in urban areas as babies died without the diagnosis having been made and treatment instituted.^25^ ^26^ Since the introduction of the programme older siblings who were previously undiagnosed have also been referred to specialist centres.

The value of a co-ordinated national approach to policy development and implementation is emphasised by the English experience. The programme developing provides a model for Europe to consider as well as other countries with significant ethnic minority populations, such as Canada. Potentially it also offers important lessons for implementation of programmes in Africa where the burden of disease is significant and where the World Health Organization is supporting the introduction of screening of newborns.^27^^–^29

In planning implementation it is important to use estimates that can be adapted as information becomes available to ensure that service capacity is put in place as appropriate. Our experience is that this may still lead to an underestimate of need – see current information available.^30^ The national approach allowed the development of national materials and training programmes that make much more efficient use of limited resources and should ensure consistency of information and messages; this was not always the case previously. The technology and screening methods are excellent compared with the standards that some screening programmes have had to live with (such as cancer screening programmes where much lower specificity and sensitivity is achieved). The programme has also driven, and continues to drive, further developments in methods.^31^ ^32^ Adaptation of equipment for the UK and ongoing developments suggest that the UK provides a good site for development of screening techniques and methods.

Evidence suggests that when carried out correctly, the sensitivity and specificity of HPLC and IEF is close to 100%, and therefore these are very reliable methods to use for a screening programme. This does not, of course, mean that there will never be a missed case. Attention to technical detail remains essential if such high standards are to be maintained after the excitement of the early days of the programme. It is important to take account of the usual age of sampling in different countries to ensure, for example, that the algorithms in the HPLC software take account of the percentage of HbA expected at different ages.

Remaining challenges, such as the best approach for a screening programme for unidentified variants and Hb Bart’s, emphasise the need for better data and research that England, with its consistent approach and increasing population, and assuming adequate resourcing, is well placed to do. This work is likely to be useful to other countries.

Those working in a laboratory may consider the technical aspects the most important but for the development of a successful programme all key stakeholders need to be engaged and development of training, counselling and clinical follow-up is crucial to the establishment of a successful programme. It goes without staying that user and voluntary sector support are important and a key reason for the success of the programme has been the multidisciplinary steering group led by a strong lay chairperson (now Archbishop of York, who is himself originally a refugee from the time of Idi Amin) to ensure that at all times the screening programme takes account of the user perspective. Implementing a programme that involves identifying a large number of genetic carriers of a recessive condition must not be undertaken as a technical exercise. Wide community engagement and support are essential. There is still a considerable way to go with improving understanding in the communities at highest risk.

## SUMMARY STATEMENT

The commitment of the UK government to introducing a linked newborn and antenatal screening programme is nearly achieved for England. A good programme is being developed that provides a model for the rest of the UK and for other countries in Europe, although challenges remain to be addressed and services for the care of affected patients require considerable development to keep up with the scale of the conditions revealed by the programme. The experience of implementing this programme could, with modification, provide a model and expertise to support developments of newborn screening programmes in Africa.
